# Fibre-Related Dietary Patterns: Socioeconomic Barriers to Adequate Fibre Intake in Polish Adolescents. A Short Report

**DOI:** 10.3390/nu9060590

**Published:** 2017-06-10

**Authors:** Beata Krusinska, Joanna Kowalkowska, Lidia Wadolowska, Justyna Weronika Wuenstel, Malgorzata Anna Slowinska, Ewa Niedzwiedzka

**Affiliations:** Department of Human Nutrition, University of Warmia and Mazury in Olsztyn, Sloneczna 45F, 10-718 Olsztyn, Poland; beata.krusinska@uwm.edu.pl (B.K.); lidia.wadolowska@uwm.edu.pl (L.W.); justyna.wuenstel@uwm.edu.pl (J.W.W.); malgorzata.slowinska@uwm.edu.pl (M.A.S.); ewa.niedzwiedzka@uwm.edu.pl (E.N.)

**Keywords:** adolescents, fibre, dietary patterns, socioeconomic status, cluster analysis

## Abstract

There is no complete explanation for the association between socioeconomic status (SES), fibre, and whole diet described by dietary patterns. The aim of this short report was to increase the understanding of adolescent dietary patterns related to fibre in their social context. A cross-sectional study was conducted involving 1176 adolescents aged 13–18 years from central and north-eastern Poland. The overall SES was composed of five single factors: place of residence, self-declared economic situation of family, self-declared economic situation of household, paternal and maternal education. The consumption frequency of nine dietary fibre sources was collected using Block’s questionnaire and was expressed in points. Fibre dietary patterns (DPs) were drawn by cluster analysis and odds ratios (ORs) adjusted for age, sex, and BMI were calculated. Three fibre-related DPs were identified: “High-fibre” (mean frequency of total fibre intake 22.7 points; range: 0–36), “Average-fibre” (17.7 points), “Low-fibre” (14.6 points). The “High-fibre” DP was characterized by a relatively higher frequency consumption of white bread, fruit, fruit or vegetable juices, potatoes, green salad and prepared vegetables, and a moderate frequency consumption of high-fibre or bran cereals and wholegrain bread compared to the “Low-fibre” DP. The “Average-fibre” DP was characterized by a relatively higher frequency consumption of wholegrain bread and high-fibre or bran cereals and a moderate frequency consumption of fruit, fruit or vegetable juices, green salad and prepared vegetables compared to the “Low-fibre” DP. Less likely to adhere to the “High-fibre” DP were adolescents with low SES (OR: 0.55, 95% CI: 0.39–0.77) or average SES (0.58, 95% CI: 0.41–0.81) in comparison with high SES (reference) as a result of elementary or secondary paternal or maternal education, rural residence, and lower household economic situation. Similar associations were found for the “Average-fibre” DP. Low and average socioeconomic status resulting from lower parents’ education, rural residence, and lower economic situation were inversely associated with achieving a relatively high fibre intake in Polish adolescents. Consuming single high-fibre foods was not sufficient to achieve a high-fibre diet in Polish adolescents. These data suggest that the consumption of a wide variety of dietary fibre sources—both relatively high-fibre and low-fibre foods—may help Polish adolescents in achieving a relatively high-fibre diet.

## 1. Introduction

Adolescents are usually not concerned enough about their nutrition [1]. In many developed countries, adolescents’ diets are characterized by a high consumption of foods rich in fat and sugar, such as fast foods, sweets, and sweetened beverages, and low consumption of foods rich in fibre, such as whole grains, fruit, vegetables, or legumes [2]. Based on data compiled by the European Food Safety Authority (EFSA) [3], the recommended fibre intake for adolescents aged 13–18 years is 19–21 g/day. Many studies have found that most adolescents have a fibre intake below the recommended level [2,4,5]. Following the nutrition guidelines [3], the main dietary sources of fibre in the human diet should be whole grains, vegetables, and fruit.

In children and adolescents, an important role in food consumption is played by the family environment, determined by the place of residence, household economic situation and related living conditions, as well as parental education [6]. In adolescence, peers and the school environment also have an impact on food consumption, which is associated with a prolonged time of being outside the home due to attending school [1]. It is well-known that socioeconomic status has an impact on the degree of variety of food consumption by young people. Low socioeconomic status makes it difficult to balance a daily diet [5].

Diet is a complex combination of consumption and nutrient intake of different food groups. For this reason, dietary patterns—as opposed to individual foods or nutrients—have lately been recommended in nutritional epidemiology [7,8]. Currently, this approach has been widely used to assess interactions between diet and other health behaviours, socioeconomic background, etc., and to examine determinants of eating patterns in public health studies [7,8].

To date, several studies investigating socioeconomic determinants of dietary intake have included nutrients, foods, and dietary patterns in adolescents [9,10,11,12,13]. There is no complete explanation for the association between socioeconomic status (SES), fibre, and whole diet described by dietary patterns. Therefore, the aim of this study was to increase the understanding of adolescent fibre dietary patterns in their social context. Overall socioeconomic status as well as single factors were considered.

## 2. Material and Methods

### 2.1. Ethical Approval

This study was approved by the Bioethics Committee of the Faculty of Medical Sciences, University of Warmia and Mazury in Olsztyn in 2010 (resolution no. 20/2010).

### 2.2. Study Design and Sample Collection

This cross-sectional study was conducted in 2010–2011 and was a part of a larger study concerning socioeconomic status, food consumption, and body weight [14,15]. Briefly, the present study involved 1176 adolescents, including 551 boys (46.9%) and 625 girls (53.1%), aged 13–18 years (Figure S1). The participants were students of middle and high schools located in central and north-eastern Poland. The respondents came from urban and rural areas. The quota sampling method was used to obtain similar numbers of respondents by age, sex, and place of residence. Details of the sample collection with the inclusion and exclusion criteria were described previously [14].

### 2.3. Socioeconomic Data

Respondents were asked about five single factors of their socioeconomic status (SES) [14,16]. To each response category, numerical values were assigned as follows (in brackets) [14]:

○ place of residence: village (1), town <100,000 inhabitants (2), city ≥100,000 inhabitants (3);
○ self-declared economic situation of family: below average (1), average (2), above average (3);
○ self-declared economic situation of household: we live very poorly—we do not have enough resources even for the cheapest food and clothing (1), we live poorly—we do not have enough resources for housing fees (2), we live modestly—we have enough resources only for food and clothing (3), we live very thriftily (4), we live relatively thriftily (5), we live very well—we can afford everything without limitations (6);
○ paternal education: elementary (1), secondary (2), high (3);
○ maternal education: elementary (1), secondary (2), high (3).

Two factors regarding the self-declared economic situation were used: (i) to fully describe participants’ economic situation because “self-declared economic situation of household” contains a more objective approach, and “self-declared economic situation of family” contains a more subjective approach referring to other families living in a similar environment, and (ii) to achieve a better balance of the number of points within the SES index. The SES index was calculated as the product of the values assigned to the individual response categories to each SES factor. The SES index (SESI) values were converted to logarithms, and tertiles of the SESI were then created to identify adolescents with low, average, and high SES. On the basis of large epidemiological studies among Polish adolescents, it was assumed that the minimum sample size in a single SES factor subgroup was 140. For this reason, for further analysis, the response categories of three single SES factors were re-categorized:

○ place of residence: “rural”, “urban” after combining two categories;
○ self-declared economic situation of the family: “average or worse” after combining two categories, “above average” (not re-categorized);
○ self-declared economic situation of household: “we live thriftily or poorly” after combining five categories, “we live very well” (not re-categorized).

Details regarding characteristics of the adolescents’ SES and its single factors have been previously reported [14].

### 2.4. Dietary Data

Data on the consumption of selected dietary fibre sources was collected by the food frequency method. In brief, a self-administered validated Block Screening Questionnaire for Fruit/Vegetable/Fibre Intake (BSQFVF) was used [17]. The BSQFVF questionnaire included nine dietary fibre sources, such as: fruit or vegetable juices, fruit (without juices), green salad, potatoes, beans, prepared vegetables (e.g., cooked, preserved, or marinated, excluding beans), high-fibre or bran cereal, wholegrain bread, white bread (including French or Italian bread, biscuits, and muffins). The frequency of food consumption was expressed in five categories to which points were assigned as follows: less than once per week (0 points), once per week (1 point), 2–3 times per week (2 points), 4–6 times per week (3 points), every day (4 points). The total fibre intake was assessed on the basis of the sum of points from the consumption frequency of nine dietary fibre sources and expressed on a scale ranging from 0 to 36 points.

### 2.5. Confounders

Variables considered to be confounding were age, sex, and body mass index (BMI). Briefly, BMI was calculated using measured weight and height and was categorized in accordance with the International Obesity Task Force (IOTF) standards [18]. A standardized BMI *z*-score (mean = 0, SD = 1) based on the authors’ own data set was also calculated [19].

### 2.6. Statistical Analysis

The fibre dietary patterns (fibre DPs) were identified a posteriori for the total sample using cluster analysis (k-means method). Input variables were the consumption frequency of nine dietary fibre sources (in points). The solution was found after four iterations. Three clusters were selected. The means and 95% confidence intervals (95% CI) were calculated for the frequency consumption of dietary fibre sources. All data were converted to logarithms before analysis. The Kruskal–Wallis test was used to compare means of the frequency of fibre intake and its selected dietary sources between fibre DPs. The distribution of the participants in relation to socioeconomic status by fibre DPs groups was also calculated. Variable distributions were compared by the Pearson Chi^2^ test with Yates’ correction when necessary. The association between socioeconomic status or its single factors and the prevalence of “Average-fibre” and “High-fibre” DPs as categorical-dependent variables was analysed using logistic regression analysis. Odds ratios (ORs) adjusted for age (as continuous variable), sex, and BMI (as categorical variables) and 95% confidence interval (95% CI) were also calculated. The reference groups were adolescents with the “Low-fibre” DP and high SES or with the highest response category of the single SES factors (OR = 1.00). The significance of ORs was assessed using Wald’s test. The statistical analysis was performed using STATISTICA statistical software (version 10.0 PL; StatSoft Inc., Tulsa, OK, USA; StatSoft, Krakow, Poland). A *p*-value < 0.05 was considered statistically significant.

## 3. Results

The distributions of the socioeconomic variables are shown in Table 1, Tables S1 and S2. The overall socioeconomic status of Polish adolescents—measured as the SES index—was the result of all socioeconomic single factors. In the low SES group, the majority of adolescents lived in a rural area (85.9%), with a lower self-declared economic situation of family (94.6%) and household (69.9%) and elementary paternal (81.2%) and maternal (72.2%) education (Table S1).

Three fibre DPs were identified: “High-fibre” (37.8% of total-sample), “Average-fibre” (24.3% of total-sample) and “Low-fibre” (37.9% of total-sample). The “High-fibre” DP was characterized by a relatively higher frequency of total fibre intake (mean 22.7 points) and higher frequency consumption of white bread, fruit, fruit or vegetable juices, potatoes, green salad, and prepared vegetables, and moderate frequency consumption of high-fibre or bran cereals and wholegrain bread compared to the “Low-fibre” DP (14.6 points) (Table 1). The “Average-fibre” DP was characterized by a relatively higher frequency of total fibre intake (17.7 points), higher frequency consumption of wholegrain bread and high-fibre or bran cereals, and moderate frequency consumption of fruit, fruit or vegetable juices, green salad, and prepared vegetables, but lower frequency consumption of white bread compared to the “Low-fibre” DP (Table 1).

Table 2 shows odds ratios of fibre dietary patterns by socioeconomic status (low/average vs. high), place of living (rural vs. urban), self-declared economic situation of family (“average or worse” vs. “above average”), self-declared economic situation of household (“we live thriftily or poorly” vs. “we live very well”), paternal and maternal education (elementary/secondary vs. high). Less likely to adhere to the “High-fibre” DP were adolescents with low SES (OR: 0.55, 95% CI: 0.39–0.77) or average SES (0.58, 95% CI: 0.41–0.81) in comparison with high SES (reference) as a result of elementary or secondary paternal (OR: 0.49, 95% CI: 0.33–0.73; OR: 0.64, 95% CI: 0.44–0.94, respectively) or maternal education (OR: 0.60, 95% CI: 0.42–0.86; 0.66, 95% CI: 0.47–0.91, respectively), rural residence (OR: 0.71, 95% CI: 0.54–0.93), and a lower economic situation of household (OR: 0.73, 95% CI: 0.56–0.95). Similar associations were seen in the “Average-fibre” DP, except for self-declared economic situation of household and secondary paternal education. There was no linear trend in ORs from low- through average-to-high-fibre-related DPs (Table 2).

Table S3 shows detailed data in regards to the frequency of fibre intake and its dietary sources, socioeconomic status, and fibre dietary patterns. Within the “High-fibre” DP, rural residence was associated with a lower frequency of consumption of fruit/vegetable juices; a lower economic situation of household or family was associated with a lower frequency consumption of fruit/vegetable juices, but with a higher frequency consumption of high-fibre or bran cereal; a lower maternal education was associated with a lower frequency consumption of fruit or fruit/vegetable juices, and a lower paternal education was associated with a lower frequency consumption of beans. Within the “Average-fibre” DP, a lower economic situation of family or household was associated with a lower frequency consumption of prepared vegetables or wholegrain bread but with a higher frequency consumption of beans, and a lower paternal education was associated with a lower frequency consumption of green salad (Table S3).

## 4. Discussion

The present study indicates that a relatively high-fibre dietary pattern was a result of the frequent consumption of a wide variety of dietary fibre sources, both relatively high-fibre and low-fibre foods. Relatively high fibre intake cannot be achieved by the frequent consumption of a single high-fibre dietary source. In addition, the “High-fibre” DP was inversely associated with a low and average socioeconomic status, including elementary and secondary parents’ education, rural residence, and a lower household economic situation of Polish adolescents. The inverse association between single socioeconomic factors and consumption frequency of dietary fibre sources was shown, even within a relatively high-fibre dietary pattern.

The present finding showed that in Polish adolescents, the “High-fibre” DP was achieved by the frequent consumption of many different sources of fibre, such as white bread, fruit, fruit or vegetable juices, potatoes, green salad, and prepared vegetables. In that context, a relatively high-fibre dietary pattern should be considered as a varied diet. Similarly, in German adolescents, the “Healthy” DP was positively correlated with the consumption of rice, legumes, vegetables, and fruit [12]. Among Italian and Spanish adolescents, the “Mediterranean” diet was characterized by a high intake of fruit and vegetables only, regarding foods containing fibre [10,13].

Polish adolescents with a low or average SES were less likely to adhere to the “High-fibre” DP (ORs = 0.55 and 0.58, respectively). Danish adolescents from low family social class were more likely to consume inadequate amounts of fruit (OR = 1.60) and vegetables (OR = 1.79) [20]. In many studies, a high family SES was positively associated with the “Mediterranean” [10] and the “Healthy” DPs [12], with adequate fibre intake [21] and the consumption of high-fibre foods such as: fruit, vegetables, and whole grains [21,22,23]. In fact, a higher family SES allows for the purchase and consumption of more expensive foods with a higher nutritional value, which are rich in fibre and have a potentially beneficial effect on health.

In the present study, the “High-fibre” DP was inversely associated with rural place of residence (OR = 0.71). In German adolescents, the “Healthy” pattern was positively associated with a higher community size [12]. In contrast, among Italian adolescents, the “Mediterranean” diet was negatively associated with living in an urban area (OR = 0.65) [10]. Differences between urban and rural area in food consumption are explained by differences in food availability. In cities in Poland and most other countries, there is a greater availability and choice of a wide range of juices, wholegrain foods, vegetables, and fruit (including exotic fruit) throughout the year. In rural stores, mainly white bread and seasonal fruit and vegetables are offered [6]. In addition, there are organizational difficulties in rural areas related to the purchase and transport of food bought in the city.

This study showed that the “High-fibre” DP was negatively associated with a lower household economic situation. In many countries, adolescents with high family income are more likely to consume fruit, vegetables, juice, and whole grain products than their peers from a low-income family [9,22,24,25,26]. Since highly nutrient-dense food such as high-fibre vegetables and fruit have a relatively high price and were associated with a high cost of dietary energy density [6,27], they are also more likely to be available in high-income families [27]. High food prices and low-income are significant barriers to a balanced diet [25]. Financial family difficulties limit the expenses of a family towards the purchase of cheaper and low nutritional value food.

As expected, a lower adherence to the “High-fibre” DP was found in adolescents with elementary and secondary parental education. Similarly, in Slovak adolescents, the infrequent consumption of fruit was associated with low parental socioeconomic position [28]. Among Norwegian adolescents [9,11,22,26], the consumption of fruit, vegetables, juice, and whole grain products increased with higher parental education. In general, parents with higher educational level have a higher nutritional knowledge and are more aware of the impact of nutrition on health and make more rational food choices in composing meals for children [22]. This finding could be explained by lower nutritional knowledge, derived mainly from the mass media and a lack of cooking skills to prepare healthy meals with the basic ingredients by parents with a lower level of education. Overall, there are two types of food-related parenting practices: restriction and pressure-to-eat, particularly among parents with elementary or secondary education level [29].

Finally, it should be underlined that associations between single socioeconomic factors and consumption frequency of dietary fibre sources were shown within fibre-related dietary patterns. Within a relatively high-fibre dietary pattern, a rural residence, a lower economic situation, and a lower parental education were especially associated with a lower consumption frequency of fruit/vegetable juices and fruits. It is well-documented that fruits are high-price, low-energy foods throughout the world, so they are less available in low-income families [27]. In Poland, fruit/vegetable juices are also high-price foods, and their consumption is limited in low-income families [16]. However, there is a strong link between poverty, low education level, and rural residence working as a vicious circle [30,31]. The next finding is related to the average-fibre dietary pattern and the lower economic situation and lower paternal education—both factors were associated with the lower-frequency consumption of green salad, prepared vegetables, and whole-grain bread. This highlights the statement referring to the economic context. Another surprising finding was that a lower economic situation was associated with the higher-frequency consumption of high-fibre or bran cereal and beans. Since these high-fibre foods are relatively cheap (especially beans), they might be introduced into the diet of people with pro-healthy attitudes but with lower incomes [32].

There are a few limitations of the present study. Firstly, the limitation of the study is a lack of quantitative data on food consumption and fibre intake. The fibre DPs were identified based on frequencies of food consumption. It could be argued that qualitative data do not exactly reflect the quantitative data. However, the fibre intake scores (in points) estimated from the validated Block’s questionnaire were compared with multi-day dietary records, and large validation studies [33] have shown good correlations with fibre intake (grams/day). Secondly, perhaps the study could benefit from the inclusion of total energy intake and energy balance into the confounding variables. However, energy intake estimated from subjective dietary assessment method such as the 24 h dietary recall is not enough to predict the usual intake because of the intra-individual variability across the consumption days, and may under- or over-report food consumption [34]. For this reason, odds ratios were adjusted for BMI, calculated on the basis of the measured weight and height. Although we made an adjustment for several confounding variables, we are not able to rule out the residual confounding bias related to other possible confounding factors (known and unknown) that were not considered in this study. Moreover, due to the cross-sectional study design, the data on each respondent were obtained at a single point in time, and only an association between a risk factor and an outcome—but not causation—can be inferred from the study [35].

The strengths of this study include a relatively large sample of more than 1000 adolescents. Since the sample was not randomly selected, the results should not be generalized to the national level, but they do provide interesting insights into overall socioeconomic status as well as its single factors and their association with dietary patterns related to fibre intake in adolescents.

## 5. Conclusions

Low and average socioeconomic status resulting from lower parents’ education, rural residence and lower economic situation were inversely associated with achieving a relatively high fibre intake in Polish adolescents. This association with single socioeconomic factors was shown even within a relatively high-fibre dietary pattern, and was expressed by the lower frequency consumption of high-price foods. Consuming a single high-fibre food was not sufficient to achieve a high-fibre diet in Polish adolescents. These data suggest that the consumption of a wide variety of dietary fibre sources—both relatively high-fibre and low-fibre foods—may help Polish adolescents in achieving a relatively high-fibre diet.

Our results suggest that to increase the consumption of dietary fibre sources, future efforts should be focused on adolescents from rural areas with lower parental education and lower economic situation. In the short-term, benefits can be achieved by nutritional education addressed to adolescents from rural areas with lower parental education. In the long-term, social welfare programs could help to improve the economic availability of high-price foods in low-income Polish families and increase the supply of high-nutritional-value foods in rural areas.

## Figures and Tables

**Table 1:** Socioeconomic status (SES) single factors and frequency of fibre intake by fibre dietary patterns (DPs) in Polish adolescents.

Characteristics	Total (*n* = 1176)		“Low-Fibre” DP (*n* = 446)	“Average-Fibre” DP (*n* = 286)	“High-Fibre” DP (*n* = 444)	*p*-Value	*p* for Trend
	*n*	% or Mean (95% CI)	% or Mean (95% CI)	% or Mean (95% CI)	% or Mean (95% CI)		
Age (years) ^a^	1176	15.9 (15.8; 16.0)	15.9 (15.7; 16.0)	16.2 (16.0; 16.3)	15.7 (15.6; 15.9)	0.0031	ns
Socioeconomic status							
low	389	33.1	38.1	26.9	32.0	0.0004	
average	414	35.2	37.4	36.4	32.2		–
high	373	31.7	24.4	36.7	35.8		
Place of residence							
rural	603	51.3	57.0	45.5	49.3	0.0058	–
urban	573	48.7	43.0	54.5	50.7		
Self-declared economic situation of family							
average or worse	965	82.1	84.5	79.4	81.3	0.1806	–
above average	211	17.9	15.5	20.6	18.7		
Self-declared economic situation of household							
we live thriftily or poorly	604	51.4	53.8	57.0	45.3	0.0035	–
we live very well	572	48.6	46.2	43.0	54.7		
Paternal education							
elementary	415	35.3	42.6	27.3	33.1	0.0001	
secondary	541	46.0	43.5	51.0	45.3		–
high	220	18.7	13.9	21.7	21.6		
Maternal education							
elementary	338	28.7	33.2	22.0	28.6	0.0020	
secondary	511	43.5	44.6	45.5	41.0		–
high	327	27.8	22.2	32.5	30.4		
BMI category ^b^							
thinnest grade 3	4	0.3	0.4	0.0	0.5	0.6442	
thinnest grade 2	6	0.5	0.4	0.3	0.7		
thinnest grade 1	76	6.5	6.5	4.2	7.9		–
normal weight	917	78.0	77.6	78.7	77.9		
overweight	159	13.5	14.1	15.4	11.7		
obesity	14	1.2	0.9	1.4	1.4		
*z*-score BMI (SD) ^c^	1176	0.0 (−0.06; 0.06)	0.01 (−0.09; 0.10)	0.19 (0.07; 0.31)	−0.13 (−0.22; −0.04)	<0.0001	ns
Total fibre (points) ^a,d^	1176	18.4 (18.1; 18.7)	14.6 (14.3; 14.9)	17.7 (17.3; 18.2)	22.7 (22.4; 23.0)	<0.001	ns
Dietary fibre sources (points) ^a,e^							
White bread	1176	3.0 (3.0; 3.1)	3.4 (3.3; 3.5)	1.6 (1.5; 1.7)	3.6 (3.5; 3.7)	<0.001	ns
Potatoes	1176	2.8 (2.8; 2.9)	2.8 (2.8; 2.9)	2.3 (2.1; 2.4)	3.1 (3.1; 3.2)	<0.001	ns
Fruit	1176	2.5 (2.5; 2.6)	2.0 (1.9; 2.1)	2.4 (2.3; 2.5)	3.2 (3.1; 3.2)	<0.001	ns
Fruit/vegetable juices	1176	2.4 (2.3; 2.5)	1.8 (1.7; 1.9)	2.1 (2.0; 2.2)	3.2 (3.1; 3.3)	<0.001	ns
Green salad	1176	2.1 (2.0; 2.1)	1.4 (1.3; 1.5)	1.9 (1.8; 2.1)	2.8 (2.7; 2.9)	<0.001	ns
Prepared vegetables	1176	1.7 (1.7; 1.8)	1.2 (1.1; 1.3)	1.8 (1.7; 1.9)	2.2 (2.1; 2.3)	<0.001	ns
High-fibre or bran cereal	1176	1.7 (1.6; 1.8)	1.0 (0.9; 1.1)	2.0 (1.9; 2.2)	2.1 (2.0; 2.2)	<0.001	ns
Wholegrain bread	1176	1.4 (1.4; 1.5)	0.6 (0.5; 0.6)	2.7(2.5; 2.8)	1.6 (1.4; 1.7)	<0.001	ns
Beans	1176	0.7 (0.6; 0.7)	0.4 (0.3; 0.4)	1.0 (0.8; 1.1)	0.9 (0.8; 0.9)	<0.001	ns

^a^ mean value and 95% confidence interval (95% CI); ^b^ body mass index (BMI) was calculated using measured weight and height and was categorized in accordance with the International Obesity Task Force (IOTF) standards [18]; ^c^ a standardized BMI *z*-score (mean = 0, SD = 1) based on the authors’ own data set was calculated [19]; ^d^ range of points: 0–36; ^e^ range of points: 0–4 (from “less than once per week” (0 points) to “every day” (4 points)); *n*—sample size; %—percentage of the sub-sample; *p*-value—level of significance assessed by chi^2^ test (categorical variables) or Kruskal–Wallis’ test (continuous variables); *p* for linear trend; ns—statistically insignificant.

**Table 2:** Odds ratios (ORs with 95% confidence interval (95% CI)) of fibre dietary patterns (DPs) by socioeconomic status and its single factors in Polish adolescents.

Characteristics		“Low-Fibre” DP (*n* = 446)	“Average-Fibre” DP (*n* = 286)		*p*-Value	“High-Fibre” DP (*n* = 444)		*p*-Value	*p* for Trend
		OR	OR	95% CI		OR	95% CI		
Socioeconomic status	high	Ref.	Ref.			Ref.	Ref.		
	average	1.00	0.62	0.43; 0.90	<0.05	0.58	0.41; 0.81	<0.01	ns
	low	1.00	0.46	0.31; 0.67	<0.0001	0.55	0.39; 0.77	<0.001	ns
Place of residence	urban	Ref.	Ref.			Ref.	Ref.		
	rural	1.00	0.64	0.48; 0.87	<0.01	0.71	0.54; 0.93	<0.05	ns
Self-declared economic situation of family	above average	Ref.	Ref.			Ref.	Ref.		
	average or worse	1.00	0.66	0.45; 0.98	<0.05	0.74	0.51; 1.05	ns	ns
Self-declared economic situation of household	we live very well	Ref.	Ref.			Ref.	Ref.		
	we live thriftily or poorly	1.00	1.14	0.85; 1.55	ns	0.73	0.56; 0.95	<0.05	ns
Paternal education	high	Ref.	Ref.			Ref.	Ref.		
	secondary	1.00	0.73	0.48; 1.12	ns	0.64	0.44; 0.94	<0.05	ns
	elementary	1.00	0.37	0.23; 0.58	<0.0001	0.49	0.33; 0.73	<0.001	ns
Maternal education	high	Ref.	Ref.			Ref.	Ref.		
	secondary	1.00	0.67	0.47; 0.97	<0.05	0.66	0.47; 0.91	<0.05	ns
	elementary	1.00	0.44	0.29; 0.66	<0.0001	0.60	0.42; 0.86	<0.01	ns

ORs were adjusted for: age (continuous variable in years), sex, and BMI (categorical variables with six categories); *n*—sample size; *p*-value—level of significance assessed by Wald’s test; *p* for linear trend; ns—statistically insignificant.

## Supplementary Materials

The following are available online at http://www.mdpi.com/2072-6643/9/6/590/s1, Figure S1: Flow chart of sample collection and study design, Table S1: Single factors of socioeconomic status by socioeconomic status categories in Polish adolescents, Table S2: Socioeconomic status and its single factors and frequency consumption of dietary fibre sources in Polish boys and girls, Table S3: The frequency (in points) of fibre intake and its dietary sources by socioeconomic status and fibre dietary patterns in Polish adolescents (mean with 95% confidence interval).
