# Supplementary material for: Fibre-Related Dietary Patterns: Socioeconomic Barriers to Adequate Fibre Intake in Polish Adolescents. A Short Report
Source: Nutrients. 2017 Jun 10;9(6):590. doi: 10.3390/nu9060590 (PMC5490569; doi:10.3390/nu9060590)
Supplement: Supplementary file 1 [file nutrients-09-00590-s001.pdf]

Supplementary Materials: Fibre-Related Dietary Patterns: Socioeconomic Barriers to Adequate Fibre Intake in Polish Adolescents. A Short Report

Beata Krusinska, Joanna Kowalkowska *, Lidia Wadolowska, Justyna Weronika Wuenstel, Malgorzata Anna Slowinska and Ewa Niedzwiedzka

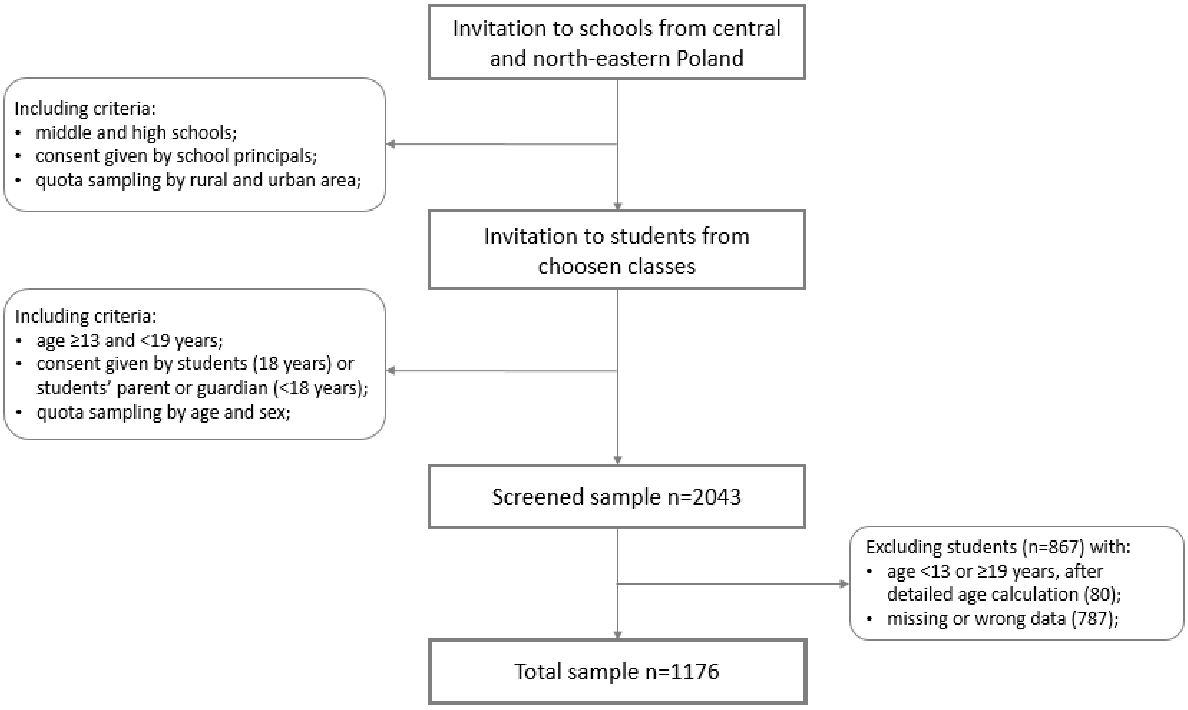

Figure S1. Flow chart of sample collection and study design

Table S1. Single factors of socioeconomic status by socioeconomic status categories in Polish adolescents

Characteristics	Total (n=1176)	Socioeconomic status Low (n=389)	Average (n=414)	High (n=373)	*p*-value
	n	%	%	%	
Place of residence					
rural	603	85.9	52.2	14.2	<0.0001
urban	573	14.1	47.8	85.8	
Self-declared economic situation of family					
average or worse	965	94.6	87.0	63.5	<0.0001
above average	211	5.4	13.0	36.5	
Self-declared economic situation of household					
we live thriftily or poorly	604	69.9	54.6	28.4	<0.0001
we live very well	572	30.1	45.4	71.6	
Paternal education					
elementary	415	81.2	22.0	2.1	
secondary	541	18.3	73.7	44.2	<0.0001
high	220	0.5	4.3	53.6	
Maternal education					
elementary	338	72.2	13.8	0.0	
secondary	511	25.2	70.3	32.7	<0.0001
high	327	2.6	15.9	67.3	

n – sample size; % – percentage of the sub-sample; *p*-value – level of significance assessed by chi^2 test.

Table S2. Socioeconomic status and its single factors and frequency consumption of dietary fibre sources in Polish boys and girls

Characteristics	Total	Boys (n=551)	Girls (n=625)	*p*-value
	n	% or mean (95%CI)	% or mean (95%CI)	
Age (years)[a]	1176	15.9 (15.7; 16.0)	15.9 (15.8; 16.0)	0.7897
Socioeconomic status				
low	389	30.3	35.5	
average	414	35.2	35.2	0.0861
high	373	34.5	29.3	
Place of residence				
rural	603	49.2	53.1	0.1973
urban	573	50.8	46.9	
Self-declared economic situation of family				
average or worse	965	77.1	86.4	0.0001
above average	211	22.9	13.6	
Self-declared economic situation of household				
we live thriftily or poorly	604	55.5	47.7	0.0085
we live very well	572	44.5	52.3	
Paternal education				
elementary	415	31.6	38.6	
secondary	541	47.7	44.5	0.0318
high	220	20.7	17.0	
Maternal education				
elementary	338	26.3	30.9	
secondary	511	43.4	43.5	0.1076
high	327	30.3	25.6	

[a]mean value and 95% confidence interval (95%CI); [b]BMI was calculated using measured weight and height and was categorized in accordance with the International Obesity Task Force (IOTF) standards [18]; [c]a standardized BMI z-score (mean=0, SD=1) based on the authors' own data set was calculated [19]; [d]range of points: 0-36; [e]range of points: 0-4 (from 'less than once per week' (0 points) to 'every day' (4 points)); n – sample size; % – percentage of the sub-sample;

p-value – level of significance assessed by chi^2 test (categorical variables) or Kruskal-Wallis' test (continuous variables).

Table S2. Continued

Characteristics	Total	Boys (n=551)	Girls (n=625)	p-value
	n	% or mean (95%CI)	% or mean (95%CI)	
BMI category[b]				
thinnest grade 3	4	0.0	0.6	<0.0001
thinnest grade 2	6	0.2	0.8	
thinnest grade 1	76	3.1	9.4	
normal weight	917	75.1	80.5	
overweight	159	19.8	8.0	
obesity	14	1.8	0.6	
z-score BMI (SD)[c]	1176	0.19 (0.10; 0.27)	-0.17 (-0.24; -0.09)	<0.0001
Total fibre (points)[ad]	1176	18.2 (17.8; 18.6)	18.6 (18.2; 19.0)	0.1371
Dietary fibre sources (points)[ae]				
White bread	1176	3.1 (3.0; 3.2)	3.0 (2.9; 3.1)	0.1220
Potatoes	1176	2.9 (2.8; 3.0)	2.8 (2.7; 2.8)	0.0671
Fruit	1176	2.4 (2.3; 2.5)	2.6 (2.5; 2.7)	0.0034
Fruit/vegetable juices	1176	2.3 (2.2; 2.4)	2.5 (2.4; 2.5)	0.1142
Green salad	1176	2.0 (1.9; 2.1)	2.2 (2.1; 2.2)	0.0180
Prepared vegetables	1176	1.7 (1.6; 1.8)	1.8 (1.7; 1.8)	0.8578
High-fibre or bran cereal	1176	1.7 (1.5; 1.8)	1.7 (1.6; 1.8)	0.1812
Wholegrain bread	1176	1.3 (1.2; 1.4)	1.6 (1.5; 1.7)	0.0031
Beans	1176	0.8 (0.7; 0.9)	0.6 (0.6; 0.7)	0.0011

[a]mean value and 95% confidence interval (95%CI); [b]BMI was calculated using measured weight and height and was categorized in accordance with the International Obesity Task Force (IOTF) standards [18]; [c]a standardized BMI z-score (mean=0, SD=1) based on the authors' own data set was calculated [19]; [d]range of points: 0-36; [e]range of points: 0-4 (from 'less than once per week' (0 points) to 'every day' (4 points)); n – sample size; % – percentage of the sub-sample; *p*-value – level of significance assessed by chi^2 test (categorical variables) or Kruskal-Wallis' test (continuous variables).

Table S3. The frequency (in points) of fibre intake and its dietary sources by socioeconomic status and fibre dietary patterns in Polish adolescents (mean with 95% confidence interval)

Dietary fibre sources	Socioeconomic status and its single factors	'Low-fibre' DP (n=446)	'Average-fibre' DP (n=286)	'High-fibre' DP (n=444)	*p*-value
Total fibre[a]	Socioeconomic status				
	low	14.2 (13.7; 14.7)	16.8 (15.9; 17.6)	22.3 (21.8; 22.9)	<0.0001
	average	14.8 (14.3; 15.3)	18.1 (17.4; 18.8)	22.7 (22.2; 23.2)	<0.0001
	high	15.0 (14.3; 15.6)	18.1 (17.4; 18.8)	23.0 (22.5; 23.6)	<0.0001
	p-value	ns	ns	ns	
	Residence				
	rural	14.5 (14.1; 14.9)	17.4 (16.8; 18.1)	22.7 (22.2; 23.1)	<0.0001
	urban	14.8 (14.3; 15.2)	18.0 (17.4; 18.5)	22.7 (22.3; 23.2)	<0.0001
	p-value	ns	ns	ns	
	Self-declared economic situation of family				
	average or worse	14.6 (14.2; 14.9)	17.5 (17.0; 18.0)	22.6 (22.3; 23.0)	<0.0001
	above average	14.9 (14.2; 15.7)	18.6 (17.6; 19.5)	23.0 (22.2; 23.8)	<0.0001
	p-value	ns	0.0487	ns	
	Self-declared economic situation of household				
	we live thriftily or poorly	14.4 (14.0; 14.9)	17.4 (16.9; 18.0)	22.5 (22.1; 22.9)	<0.0001
	we live very well	14.8 (14.4; 15.3)	18.1 (17.4; 18.8)	22.8 (22.4; 23.3)	<0.0001
	p-value	ns	ns	ns	
	Paternal education				
	elementary	14.4 (13.9; 14.9)	17.2 (16.3; 18.2)	22.3 (21.7; 22.8)	<0.0001
	secondary	14.8 (14.3; 15.2)	17.7 (17.1; 18.3)	22.7 (22.3; 23.1)	<0.0001
	high	14.8 (14.0; 15.6)	18.4 (17.6; 19.2)	23.3 (22.5; 24.1)	<0.0001
	p-value	ns	ns	ns	

[a]range of points: 0-36; [b]range of points: 0-4 (from 'less than once per week' (0 point) to 'every day' (4 points)); n – sample size; *p*-value – level of significance

assessed by Kruskal-Wallis' test.

Table S3. Continued

**Dietary fibre sources**	**Socioeconomic status and its single factors**	**'Low-fibre' DP (n=446)**	**'Average-fibre' DP (n=286)**	**'High-fibre' DP (n=444)**	***p*-value**
Total fibre[a]	Maternal education				
	elementary	14.6 (14.0; 15.1)	16.9 (16.0; 17.9)	22.6 (22.0; 23.1)	<0.0001
	secondary	14.5 (14.1; 15.0)	17.7 (17.0; 18.3)	22.3 (21.9; 22.8)	<0.0001
	high	14.9 (14.2; 15.5)	18.3 (17.6; 19.1)	23.3 (22.7; 23.9)	<0.0001
	p-value	ns	ns	ns	
White bread[b]	Socioeconomic status				
	low	3.3 (3.1; 3.4)	1.6 (1.4; 1.9)	3.6 (3.5; 3.7)	<0.0001
	average	3.5 (3.4; 3.6)	1.6 (1.4; 1.8)	3.7 (3.5; 3.8)	<0.0001
	high	3.4 (3.2; 3.5)	1.5 (1.3; 1.8)	3.5 (3.4; 3.6)	<0.0001
	p-value	ns	ns	ns	
	Residence				
	rural	3.3 (3.2; 3.5)	1.6 (1.5; 1.8)	3.6 (3.5; 3.7)	<0.0001
	urban	3.4 (3.3; 3.5)	1.5 (1.4; 1.7)	3.6 (3.5; 3.7)	<0.0001
	p-value	ns	ns	ns	
	Self-declared economic situation of family				
	average or worse	3.4 (3.3; 3.5)	1.6 (1.5; 1.8)	3.6 (3.5; 3.7)	<0.0001
	above average	3.4 (3.2; 3.7)	1.4 (1.1; 1.7)	3.5 (3.3; 3.7)	<0.0001
	p-value	ns	ns	ns	
	Self-declared economic situation of household				
	we live thriftily or poorly	3.3 (3.2; 3.4)	1.5 (1.4; 1.7)	3.6 (3.5; 3.7)	<0.0001
	we live very well	3.5 (3.3; 3.6)	1.6 (1.4; 1.8)	3.6 (3.5; 3.7)	<0.0001
	p-value	0.0347	ns	ns	

[a]range of points: 0-36; [b]range of points: 0-4 (from 'less than once per week' (0 point) to 'every day' (4 points)); n – sample size; *p*-value – level of significance assessed by Kruskal-Wallis' test.

Table S3. Continued

Dietary fibre sources	Socioeconomic status and its single factors	'Low-fibre' DP (n=446)	'Average-fibre' DP (n=286)	'High-fibre' DP (n=444)	*p*-value
White bread[b]	Paternal education				
	elementary	3.3 (3.2; 3.5)	1.6 (1.3; 1.8)	3.6 (3.5; 3.7)	<0.0001
	secondary	3.4 (3.3; 3.5)	1.6 (1.4; 1.8)	3.6 (3.5; 3.7)	<0.0001
	high	3.5 (3.3; 3.7)	1.5 (1.3; 1.8)	3.6 (3.4; 3.7)	<0.0001
	p-value	ns	ns	ns	
	Maternal education				
	elementary	3.3 (3.2; 3.5)	1.6 (1.3; 1.9)	3.6 (3.5; 3.7)	<0.0001
	secondary	3.4 (3.3; 3.5)	1.6 (1.4; 1.8)	3.6 (3.5; 3.7)	<0.0001
	high	3.4 (3.3; 3.6)	1.6 (1.3; 1.8)	3.6 (3.4; 3.7)	<0.0001
	p-value	ns	ns	ns	
Potatoes[b]	Socioeconomic status				
	low	2.8 (2.7; 3.0)	2.2 (1.9; 2.5)	3.1 (3.0; 3.3)	<0.0001
	average	2.9 (2.8; 3.1)	2.4 (2.1; 2.6)	3.3 (3.1; 3.4)	<0.0001
	high	2.8 (2.6; 3.0)	2.2 (2.0; 2.4)	3.1 (2.9; 3.2)	<0.0001
	p-value	ns	ns	ns	
	Residence				
	rural	2.9 (2.8; 3.0)	2.3 (2.1; 2.5)	3.1 (3.0; 3.3)	<0.0001
	urban	2.8 (2.6; 2.9)	2.3 (2.1; 2.4)	3.2 (3.0; 3.3)	<0.0001
	p-value	ns	ns	ns	
	Self-declared economic situation of family				
	average or worse	2.8 (2.7; 2.9)	2.2 (2.1; 2.3)	3.1 (3.0; 3.2)	<0.0001
	above average	2.9 (2.6; 3.1)	2.5 (2.2; 2.8)	3.2 (3.0; 3.4)	0.0008
	p-value	ns	ns	ns	

[a]range of points: 0-36; [b]range of points: 0-4 (from 'less than once per week' (0 point) to 'every day' (4 points)); n – sample size; *p*-value – level of significance assessed by Kruskal-Wallis' test.

Table S3. Continued

Dietary fibre sources	Socioeconomic status and its single factors	'Low-fibre' DP (n=446)	'Average-fibre' DP (n=286)	'High-fibre' DP (n=444)	*p*-value
Potatoes[b]	Self-declared economic situation of household				
	we live thriftily or poorly	2.9 (2.7; 3.0)	2.3 (2.1; 2.5)	3.2 (3.1; 3.3)	<0.0001
	we live very well	2.8 (2.7; 3.0)	2.2 (2.0; 2.4)	3.1 (3.0; 3.2)	<0.0001
	p-value	ns	ns	ns	
	Paternal education				
	elementary	2.8 (2.7; 3.0)	2.2 (1.9; 2.5)	3.2 (3.1; 3.3)	<0.0001
	secondary	2.9 (2.8; 3.1)	2.3 (2.1; 2.5)	3.1 (3.0; 3.2)	<0.0001
	high	2.8 (2.5; 3.0)	2.3 (2.0; 2.6)	3.2 (3.0; 3.4)	<0.0001
	p-value	ns	ns	ns	
	Maternal education				
	elementary	2.9 (2.7; 3.0)	2.3 (2.0; 2.6)	3.2 (3.1; 3.4)	<0.0001
	secondary	2.9 (2.8; 3.1)	2.3 (2.1; 2.5)	3.1 (2.9; 3.2)	<0.0001
	high	2.7 (2.4; 2.9)	2.2 (2.0; 2.5)	3.2 (3.0; 3.3)	<0.0001
	p-value	ns	ns	ns	
Fruit[b]	Socioeconomic status				
	low	1.9 (1.8; 2.1)	2.2 (1.9; 2.4)	3.1 (2.9; 3.2)	<0.0001
	average	1.9 (1.8; 2.1)	2.5 (2.3; 2.7)	3.2 (3.0; 3.3)	<0.0001
	high	2.1 (1.9; 2.3)	2.5 (2.2; 2.7)	3.2 (3.1; 3.3)	<0.0001
	p-value	ns	ns	ns	
	Residence				
	rural	1.9 (1.8; 2.1)	2.3 (2.1; 2.5)	3.2 (3.1; 3.3)	<0.0001
	urban	2.0 (1.8; 2.1)	2.4 (2.3; 2.6)	3.1 (3.0; 3.3)	<0.0001
	p-value	ns	ns	ns	

[a]range of points: 0-36; [b]range of points: 0-4 (from 'less than once per week' (0 point) to 'every day' (4 points)); n – sample size; *p*-value – level of significance assessed by Kruskal-Wallis' test.

Table S3. Continued

Dietary fibre sources	Socioeconomic status and its single factors	'Low-fibre' DP (n=446)	'Average-fibre' DP (n=286)	'High-fibre' DP (n=444)	*p*-value
Fruit[b]	Self-declared economic situation of family				
	average or worse	2.0 (1.9; 2.1)	2.4 (2.2; 2.5)	3.1 (3.0; 3.2)	<0.0001
	above average	2.0 (1.8; 2.3)	2.5 (2.2; 2.7)	3.2 (3.1; 3.4)	<0.0001
	p-value	ns	ns	ns	
	Self-declared economic situation of household				
	we live thriftily or poorly	1.9 (1.8; 2.1)	2.3 (2.2; 2.5)	3.1 (3.0; 3.3)	<0.0001
	we live very well	2.0 (1.9; 2.2)	2.5 (2.2; 2.7)	3.2 (3.1; 3.3)	<0.0001
	p-value	ns	ns	ns	
	Paternal education				
	elementary	2.0 (1.9; 2.2)	2.3 (2.1; 2.6)	3.1 (3.0; 3.3)	<0.0001
	secondary	1.9 (1.7; 2.0)	2.4 (2.2; 2.6)	3.1 (3.0; 3.2)	<0.0001
	high	2.1 (1.8; 2.4)	2.5 (2.2; 2.8)	3.3 (3.1; 3.4)	<0.0001
	p-value	ns	ns	ns	
	Maternal education				
	elementary	2.0 (1.8; 2.1)	2.2 (2.0; 2.5)	3.1 (2.9; 3.2)	<0.0001
	secondary	2.0 (1.8; 2.1)	2.4 (2.2; 2.6)	3.1 (3.0; 3.2)	<0.0001
	high	2.0 (1.8; 2.2)	2.5 (2.3; 2.8)	3.3 (3.1; 3.4)	<0.0001
	p-value	ns	ns	0.0455	
Fruit/vegetable juices[b]	Socioeconomic status				
	low	1.7 (1.5; 1.9)	2.0 (1.7; 2.3)	3.1 (2.9; 3.2)	<0.0001
	average	1.8 (1.7; 2.0)	2.2 (2.0; 2.4)	3.1 (3.0; 3.3)	<0.0001
	high	1.9 (1.6; 2.1)	2.1 (1.9; 2.3)	3.4 (3.3; 3.5)	<0.0001
	p-value	ns	ns	0.0021	

[a]range of points: 0-36; [b]range of points: 0-4 (from 'less than once per week' (0 point) to 'every day' (4 points)); n – sample size; *p*-value – level of significance assessed by Kruskal-Wallis' test.

Table S3. **Continued**

Dietary fibre sources	Socioeconomic status and its single factors	'Low-fibre' DP (n=446)	'Average-fibre' DP (n=286)	'High-fibre' DP (n=444)	*p*-value
Fruit/vegetable juices[b]	Residence				
	rural	1.8 (1.6; 1.9)	2.0 (1.8; 2.2)	3.1 (3.0; 3.2)	<0.0001
	urban	1.8 (1.7; 2.0)	2.2 (2.0; 2.4)	3.3 (3.2; 3.4)	<0.0001
	p-value	ns	ns	0.0206	
	Self-declared economic situation of family				
	average or worse	1.8 (1.7; 1.9)	2.1 (1.9; 2.2)	3.1 (3.1; 3.2)	<0.0001
	above average	1.9 (1.6; 2.2)	2.2 (1.9; 2.5)	3.5 (3.3; 3.6)	<0.0001
	p-value	ns	ns	0.0041	
	Self-declared economic situation of household				
	we live thriftily or poorly	1.7 (1.6; 1.9)	2.1 (1.9; 2.2)	3.0 (2.9; 3.2)	<0.0001
	we live very well	1.9 (1.7; 2.0)	2.1 (1.9; 2.4)	3.3 (3.2; 3.4)	<0.0001
	p-value			0.0006	
	Paternal education				
	elementary	1.8 (1.6; 1.9)	2.2 (1.9; 2.4)	3.1 (3.0; 3.3)	<0.0001
	secondary	1.8 (1.7; 2.0)	2.1 (1.9; 2.2)	3.2 (3.1; 3.4)	<0.0001
	high	1.8 (1.5; 2.2)	2.1 (1.8; 2.4)	3.3 (3.1; 3.5)	<0.0001
	p-value	ns	ns	ns	
	Maternal education				
	elementary	1.7 (1.6; 1.9)	1.8 (1.6; 2.1)	3.1 (3.0; 3.3)	<0.0001
	secondary	1.8 (1.7; 2.0)	2.2 (2.0; 2.4)	3.1 (3.0; 3.3)	<0.0001
	high	1.8 (1.6; 2.1)	2.1 (1.9; 2.3)	3.4 (3.3; 3.6)	<0.0001
	p-value	ns	ns	0.0035	

[a]range of points: 0-36; [b]range of points: 0-4 (from 'less than once per week' (0 point) to 'every day' (4 points)); n – sample size; *p*-value – level of significance assessed by Kruskal-Wallis' test.

Table S3. **Continued**

Dietary fibre sources	Socioeconomic status and its single factors	'Low-fibre' DP (n=446)	'Average-fibre' DP (n=286)	'High-fibre' DP (n=444)	*p*-value
Green salad[b]	Socioeconomic status				
	low	1.4 (1.2; 1.5)	1.7 (1.4; 1.9)	2.8 (2.6; 2.9)	<0.0001
	average	1.4 (1.2; 1.6)	1.9 (1.7; 2.1)	2.8 (2.6; 3.0)	<0.0001
	high	1.5 (1.4; 1.7)	2.1 (1.9; 2.4)	2.9 (2.7; 3.0)	<0.0001
	p-value	ns	0.0415	ns	
	Residence				
	rural	1.4 (1.3; 1.6)	1.8 (1.6; 2.0)	2.8 (2.6; 2.9)	<0.0001
	urban	1.4 (1.3; 1.6)	2.0 (1.8; 2.2)	2.9 (2.7; 3.0)	<0.0001
	p-value	ns	ns	ns	
	Self-declared economic situation of family				
	average or worse	1.4 (1.3; 1.5)	1.9 (1.7; 2.0)	2.8 (2.7; 2.9)	<0.0001
	above average	1.4 (1.2; 1.7)	2.1 (1.8; 2.4)	2.7 (2.5; 3.0)	<0.0001
	p-value	ns	ns	ns	
	Self-declared economic situation of household				
	we live thriftily or poorly	1.4 (1.2; 1.5)	1.9 (1.7; 2.1)	2.8 (2.7; 2.9)	<0.0001
	we live very well	1.5 (1.4; 1.7)	2.0 (1.8; 2.2)	2.8 (2.7; 3.0)	<0.0001
	p-value	ns	ns	ns	
	Paternal education				
	elementary	1.4 (1.3; 1.5)	1.7 (1.5; 2.0)	2.8 (2.7; 3.0)	<0.0001
	secondary	1.4 (1.3; 1.6)	1.8 (1.7; 2.0)	2.8 (2.7; 3.0)	<0.0001
	high	1.5 (1.2; 1.8)	2.4 (2.1; 2.6)	2.8 (2.5; 3.0)	<0.0001
	p-value	ns	0.0055	ns	

[a]range of points: 0-36; [b]range of points: 0-4 (from 'less than once per week' (0 point) to 'every day' (4 points)); n – sample size; *p*-value – level of significance assessed by Kruskal-Wallis' test.

Table S3. **Continued**

Dietary fibre sources	Socioeconomic status and its single factors	'Low-fibre' DP (n=446)	'Average-fibre' DP (n=286)	'High-fibre' DP (n=444)	*p*-value
Green salad[b]	Maternal education				
	elementary	1.5 (1.3; 1.6)	1.8 (1.5; 2.0)	2.7 (2.6; 2.9)	<0.0001
	secondary	1.4 (1.3; 1.6)	1.9 (1.7; 2.1)	2.8 (2.7; 2.9)	<0.0001
	high	1.4 (1.2; 1.6)	2.1 (1.8; 2.3)	2.9 (2.7; 3.1)	<0.0001
	p-value	ns	ns	ns	
Prepared vegetables[b]	Socioeconomic status				
	low	1.3 (1.1; 1.5)	1.6 (1.4; 1.8)	2.2 (2.0; 2.4)	<0.0001
	average	1.2 (1.1; 1.3)	1.8 (1.6; 2.0)	2.1 (1.9; 2.3)	<0.0001
	high	1.1 (1.0; 1.3)	1.9 (1.7; 2.1)	2.4 (2.2; 2.6)	<0.0001
	p-value	ns	ns	ns	
	Residence				
	rural	1.3 (1.1; 1.4)	1.8 (1.6; 2.0)	2.3 (2.1; 2.4)	<0.0001
	urban	1.2 (1.0; 1.3)	1.8 (1.6; 1.9)	2.2 (2.1; 2.4)	<0.0001
	p-value	ns	ns	ns	
	Self-declared economic situation of family				
	average or worse	1.2 (1.1; 1.3)	1.7 (1.6; 1.9)	2.2 (2.1; 2.3)	<0.0001
	above average	1.2 (1.0; 1.5)	2.1 (1.8; 2.3)	2.4 (2.2; 2.7)	<0.0001
	p-value	ns	0.0209	ns	
	Self-declared economic situation of household				
	we live thriftily or poorly	1.2 (1.1; 1.3)	1.7 (1.5; 1.8)	2.2 (2.0; 2.3)	<0.0001
	we live very well	1.2 (1.1; 1.4)	1.9 (1.7; 2.1)	2.3 (2.2; 2.4)	<0.0001
	p-value	ns	ns	ns	

[a]range of points: 0-36; [b]range of points: 0-4 (from 'less than once per week' (0 point) to 'every day' (4 points)); n – sample size; *p*-value – level of significance assessed by Kruskal-Wallis' test.

Table S3. **Continued**

Dietary fibre sources	Socioeconomic status and its single factors	'Low-fibre' DP (n=446)	'Average-fibre' DP (n=286)	'High-fibre' DP (n=444)	*p*-value
Prepared vegetables[b]	Paternal education				
	elementary	1.3 (1.1; 1.4)	1.5 (1.3; 1.8)	2.1 (1.9; 2.3)	<0.0001
	secondary	1.2 (1.1; 1.3)	1.9 (1.7; 2.1)	2.2 (2.1; 2.4)	<0.0001
	high	1.1 (0.9; 1.4)	1.9 (1.6; 2.1)	2.4 (2.2; 2.7)	<0.0001
	p-value	ns	ns	ns	
	Maternal education				
	elementary	1.2 (1.0; 1.4)	1.6 (1.4; 1.9)	2.2 (2.0; 2.4)	<0.0001
	secondary	1.2 (1.1; 1.3)	1.8 (1.6; 1.9)	2.2 (2.1; 2.4)	<0.0001
	high	1.2 (1.0; 1.4)	1.9 (1.7; 2.2)	2.3 (2.1; 2.5)	<0.0001
	p-value	ns	ns	ns	
High-fibre or bran cereal[b]	Socioeconomic status				
	low	1.0 (0.8; 1.1)	2.0 (1.7; 2.3)	2.1 (2.0; 2.3)	<0.0001
	average	1.0 (0.9; 1.2)	2.2 (1.9; 2.4)	2.2 (2.0; 2.4)	<0.0001
	high	1.2 (1.0; 1.4)	2.0 (1.7; 2.2)	2.0 (1.9; 2.2)	<0.0001
	p-value	ns	ns	ns	
	Residence				
	rural	1.0 (0.8; 1.1)	2.0 (1.8; 2.2)	2.2 (2.0; 2.3)	<0.0001
	urban	1.2 (1.0; 1.3)	2.1 (1.9; 2.3)	2.1 (1.9; 2.2)	<0.0001
	p-value	0.0302	ns	ns	
	Self-declared economic situation of family				
	average or worse	1.0 (0.9; 1.1)	2.0 (1.9; 2.2)	2.2 (2.1; 2.3)	<0.0001
	above average	1.1 (0.8; 1.4)	2.1 (1.8; 2.4)	1.8 (1.5; 2.1)	<0.0001
	p-value	ns	ns	0.0056	

[a]range of points: 0-36; [b]range of points: 0-4 (from 'less than once per week' (0 point) to 'every day' (4 points)); n – sample size; *p*-value – level of significance

assessed by Kruskal-Wallis' test.

Table S3. Continued

**Dietary fibre sources**	**Socioeconomic status and its single factors**	**'Low-fibre' DP (n=446)**	**'Average-fibre' DP (n=286)**	**'High-fibre' DP (n=444)**	***p*-value**
High-fibre or bran cereal[b]	Self-declared economic situation of household				
	we live thriftily or poorly	1.1 (0.9; 1.2)	2.0 (1.8; 2.2)	2.2 (2.0; 2.3)	<0.0001
	we live very well	1.0 (0.9; 1.2)	2.1 (1.9; 2.4)	2.1 (1.9; 2.2)	<0.0001
	p-value	ns	ns	ns	
	Paternal education				
	elementary	0.9 (0.8; 1.1)	2.1 (1.8; 2.4)	2.1 (1.9; 2.3)	<0.0001
	secondary	1.1 (1.0; 1.3)	2.1 (1.9; 2.3)	2.2 (2.0; 2.3)	<0.0001
	high	1.1 (0.8; 1.5)	1.9 (1.5; 2.2)	2.1 (1.8; 2.3)	0.0001
	p-value	ns	ns	ns	
	Maternal education				
	elementary	1.1 (0.9; 1.2)	2.1 (1.8; 2.4)	2.2 (2.0; 2.4)	<0.0001
	secondary	0.9 (0.8; 1.0)	2.0 (1.8; 2.2)	2.1 (1.9; 2.3)	<0.0001
	high	1.3 (1.0; 1.5)	2.0 (1.8; 2.3)	2.1 (1.9; 2.3)	<0.0001
	p-value	0.0471	ns	ns	
Wholegrain bread[b]	Socioeconomic status				
	low	0.5 (0.4; 0.6)	2.6 (2.3; 2.8)	1.5 (1.3; 1.7)	<0.0001
	average	0.6 (0.5; 0.7)	2.7 (2.5; 2.9)	1.5 (1.3; 1.7)	<0.0001
	high	0.6 (0.4; 0.7)	2.8 (2.6; 3.0)	1.6 (1.4; 1.8)	<0.0001
	p-value	ns	ns	ns	
	Residence				
	rural	0.5 (0.4; 0.6)	2.6 (2.4; 2.8)	1.6 (1.4; 1.7)	<0.0001
	urban	0.6 (0.5; 0.7)	2.7 (2.6; 2.9)	1.5 (1.4; 1.7)	<0.0001
	p-value	ns	ns	ns	

[a]range of points: 0-36; [b]range of points: 0-4 (from 'less than once per week' (0 point) to 'every day' (4 points)); n – sample size; *p*-value – level of significance

assessed by Kruskal-Wallis' test.

Table S3. **Continued**

Dietary fibre sources	Socioeconomic status and its single factors	'Low-fibre' DP (n=446)	'Average-fibre' DP (n=286)	'High-fibre' DP (n=444)	*p*-value
Wholegrain bread[b]	Self-declared economic situation of family				
	average or worse	0.5 (0.5; 0.6)	2.7 (2.5; 2.8)	1.5 (1.4; 1.7)	<0.0001
	above average	0.6 (0.4; 0.8)	2.7 (2.4; 3.0)	1.7 (1.4; 2.0)	<0.0001
	p-value	ns	ns	ns	
	Self-declared economic situation of household				
	we live thriftily or poorly	0.5 (0.5; 0.6)	2.6 (2.4; 2.7)	1.5 (1.4; 1.7)	<0.0001
	we live very well	0.6 (0.5; 0.7)	2.8 (2.6; 3.0)	1.6 (1.4; 1.7)	<0.0001
	p-value	ns	0.0320	ns	
	Paternal education				
	elementary	0.5 (0.4; 0.6)	2.7 (2.5; 3.0)	1.4 (1.2; 1.6)	<0.0001
	secondary	0.6 (0.5; 0.8)	2.6 (2.4; 2.8)	1.5 (1.3; 1.7)	<0.0001
	high	0.5 (0.3; 0.7)	2.8 (2.6; 3.1)	1.8 (1.5; 2.1)	<0.0001
	p-value	ns	ns	ns	
	Maternal education				
	elementary	0.6 (0.5; 0.7)	2.5 (2.2; 2.8)	1.5 (1.3; 1.7)	<0.0001
	secondary	0.5 (0.4; 0.6)	2.7 (2.5; 2.9)	1.5 (1.3; 1.6)	<0.0001
	high	0.6 (0.5; 0.8)	2.8 (2.5; 3.0)	1.7 (1.5; 1.9)	<0.0001
	p-value	ns	ns	ns	
Beans[b]	Socioeconomic status				
	low	0.4 (0.3; 0.5)	0.9 (0.7; 1.2)	0.8 (0.6; 0.9)	<0.0001
	average	0.4 (0.3; 0.5)	0.9 (0.7; 1.1)	0.9 (0.7; 1.0)	<0.0001
	high	0.4 (0.3; 0.5)	1.0 (0.8; 1.2)	0.9 (0.7; 1.0)	<0.0001
	p-value	ns	ns	ns	

[a]range of points: 0-36; [b]range of points: 0-4 (from 'less than once per week' (0 point) to 'every day' (4 points)); n – sample size; *p*-value – level of significance

assessed by Kruskal-Wallis' test.

Table S3. Continued

**Dietary fibre sources**	**Socioeconomic status and its single factors**	**'Low-fibre' DP (n=446)**	**'Average-fibre' DP (n=286)**	**'High-fibre' DP (n=444)**	***p*-value**
Beans[b]	Residence				
	rural	0.4 (0.3; 0.4)	1.0 (0.8; 1.1)	0.8 (0.7; 0.9)	<0.0001
	urban	0.4 (0.3; 0.5)	0.9 (0.8; 1.1)	0.9 (0.8; 1.0)	<0.0001
	p-value	ns	ns	ns	
	Self-declared economic situation of family				
	average or worse	0.4 (0.3; 0.4)	0.9 (0.8; 1.1)	0.8 (0.7; 0.9)	<0.0001
	above average	0.3 (0.2; 0.5)	1.0 (0.7; 1.3)	0.9 (0.7; 1.2)	<0.0001
	p-value	ns	ns	ns	
	Self-declared economic situation of household				
	we live thriftily or poorly	0.4 (0.3; 0.5)	1.1 (0.9; 1.2)	0.9 (0.7; 1.0)	<0.0001
	we live very well	0.4 (0.3; 0.5)	0.8 (0.6; 0.9)	0.8 (0.7; 1.0)	<0.0001
	p-value	ns	0.0017	ns	
	Paternal education				
	elementary	0.3 (0.2; 0.4)	0.9 (0.7; 1.1)	0.7 (0.6; 0.9)	<0.0001
	secondary	0.4 (0.3; 0.5)	0.9 (0.7; 1.1)	0.9 (0.8; 1.0)	<0.0001
	high	0.4 (0.2; 0.5)	1.2 (0.9; 1.4)	0.9 (0.7; 1.1)	<0.0001
	p-value	ns	ns	0.0494	
	Maternal education				
	elementary	0.3 (0.2; 0.4)	0.9 (0.7; 1.2)	0.9 (0.8; 1.1)	<0.0001
	secondary	0.4 (0.3; 0.5)	0.9 (0.7; 1.0)	0.8 (0.7; 0.9)	<0.0001
	high	0.4 (0.2; 0.5)	1.1 (0.9; 1.3)	0.8 (0.7; 1.0)	<0.0001
	p-value	ns	ns	ns	

[a]range of points: 0-36; [b]range of points: 0-4 (from 'less than once per week' (0 point) to 'every day' (4 points)); n – sample size; *p*-value – level of significance assessed by Kruskal-Wallis' test.
